# Phanerozoic parasitism and marine metazoan diversity: dilution versus amplification

**DOI:** 10.1098/rstb.2020.0366

**Published:** 2021-11-08

**Authors:** Kenneth De Baets, John Warren Huntley, Daniele Scarponi, Adiël A. Klompmaker, Aleksandra Skawina

**Affiliations:** ^1^ GeoZentrum Nordbayern, Fachgruppe PaläoUmwelt, Friedrich-Alexander-University Erlangen-Nürnberg, Loewenichstraße 28, 91054 Erlangen, Germany; ^2^ Department of Geological Sciences, University of Missouri, 101 Geological Sciences Building, Columbia, MO 65211, USA; ^3^ Dipartimento di Scienze Biologiche, Geologiche e Ambientali, University of Bologna, Piazza di Porta San Donato 1, 40131 Bologna, Italy; ^4^ Department of Museum Research and Collections and Alabama Museum of Natural History, University of Alabama, Box 870340, Tuscaloosa, AL 35487, USA; ^5^ Department of Animal Physiology, Faculty of Biology, University of Warsaw, Warszawa, Poland

**Keywords:** paleoparasitology, biodiversity, Metazoa, paleopathology, Phanerozoic, marine disease

## Abstract

Growing evidence suggests that biodiversity mediates parasite prevalence. We have compiled the first global database on occurrences and prevalence of marine parasitism throughout the Phanerozoic and assess the relationship with biodiversity to test if there is support for amplification or dilution of parasitism at the macroevolutionary scale. Median prevalence values by era are 5% for the Paleozoic, 4% for the Mesozoic, and a significant increase to 10% for the Cenozoic. We calculated period-level shareholder quorum sub-sampled (SQS) estimates of mean sampled diversity, three-timer (3T) origination rates, and 3T extinction rates for the most abundant host clades in the Paleobiology Database to compare to both occurrences of parasitism and the more informative parasite prevalence values. Generalized linear models (GLMs) of parasite occurrences and SQS diversity measures support both the amplification (all taxa pooled, crinoids and blastoids, and molluscs) and dilution hypotheses (arthropods, cnidarians, and bivalves). GLMs of prevalence and SQS diversity measures support the amplification hypothesis (all taxa pooled and molluscs). Though likely scale-dependent, parasitism has increased through the Phanerozoic and clear patterns primarily support the amplification of parasitism with biodiversity in the history of life.

This article is part of the theme issue ‘Infectious disease macroecology: parasite diversity and dynamics across the globe’.

## 1. Introduction

How have biotic interactions and biodiversity related to one another through the history of life? This question has been a fundamental topic of research since Darwin articulated natural selection in 1859. Numerous studies have indicated the importance and complexities of antagonistic interactions in maintaining or promoting diversity over ecological time scales and a variety of spatial scales [1–7]. How these biotic interactions scale up to influence macroevolutionary trends has been discussed extensively in the literature and Hembry & Weber [8] and Fraser *et al*. [9] have provided timely reviews of the history of and recent advances in answering such questions. Defining the distribution in time and space and the intensity of antagonistic biotic interactions as well as assessing their evolutionary implications has been a prominent theme in palaeobiological research of the marine invertebrate fossil record over the last four decades. Predator–prey interactions have received the lion's share of attention, which has highlighted the escalating intensity of predation through the Phanerozoic that may have shaped some macroevolutionary trends [10–21]. Huntley & Kowalewski's [14] compilation of predation frequencies among marine invertebrates was positively correlated with Sepkoski's [22,23] estimate of global genus-level diversity of marine animals throughout the Phanerozoic. They suggested three end-member explanations for the pattern including a causative linkage between biotic interactions and diversity over geologic time scales, the passive diffusion of predation complexity with increasing diversity, and the spurious result of sampling artefacts.

Parasitism is also pervasive in modern marine ecosystems [24,25], but has received far less attention than predation in the fossil record [24]. Among well-studied animal groups, all species host parasites and upwards of 40% of described species are parasitic [26]. Nevertheless, the evolutionary history of parasitism remains poorly constrained [24,27–29]. This fact is not surprising as the fossilization potential of soft-bodied parasites is low and they are often small, which has resulted in a patchy fossil record [30,31]. Host organisms with decay-resistant tissues, however, have a more continuous and homogeneous fossil record and provide a unique window on the evolution of parasitic relationships in the form of characteristic traces or, more rarely, direct associations with their skeletonized parasites [32,33]. Although the fossil record of unicellular pathogens is low, 80% (12/15) of metazoan parasitic interactions with their bivalve hosts have a fossilization potential and at least 53% (8/15) have been regularly reported [34]. The maximum estimates for the appearance of metazoan parasites lie in the latest Precambrian when their animal hosts first appeared [33], but, as parasitism is derived, it most likely appeared later. Metazoan fossils document the appearance of predation in the terminal Ediacaran Period [35–37], and the appearance of the earliest parasitic relationships are preserved slightly later among Cambrian brachiopods [38–41] during the explosive radiation of animal body plans [42,43]. Presumably, the occurrence and prevalence of parasitic interactions have increased since the Early Paleozoic [32,44,45], but this assumption has not been extensively tested across host taxonomic groups and geologic time. Predation is a common evolutionary pathway to parasitism [28] and we might expect to find a similar positive relationship between parasitism and biodiversity as for predation [46].

Two hypotheses have been proposed regarding the relationship between parasite diversity and biodiversity in living communities. The amplification hypothesis predicts that the evolutionary accumulation of parasite–host interactions is positively correlated with biodiversity and has resulted in increasingly complex life cycles and interlinked food webs [47–49]. Kamiya *et al*.'s [50] meta-analysis of parasite–host interactions in modern ecosystems, including multiple phyla of hosts and parasites from a broad range of spatial scales of observation, found significant support for a positive correlation between parasite diversity and host diversity. For the fossil record, Baumiller & Gahn's [32] survey of parasitic interactions through the Phanerozoic suggested a positive correlation between the number of parasitism occurrences and diversity of Paleozoic echinoderms and, possibly more generally, marine animal diversity.

Conversely, the dilution (or decoy) hypothesis, documented in many modern ecosystems [51], predicts a negative correlation between diversity and prevalence of parasitism. The dilution hypothesis was first articulated through the analysis of Lyme disease, an infection by the bacterium *Borrelia burgdorferi* that is transmitted from its reservoir hosts (a variety of mammals) through its vector, the black-legged tick (*Ixodes scapularis*), to humans. The ability of a reservoir host to transmit the pathogen to a vector is known as reservoir competence, which varies among host species. The central tenet of the dilution hypothesis, as described by Schmidt & Ostfeld [51], is that host communities with a high species richness and/or evenness will experience lower prevalence of parasitic infection because they have a higher proportion of low reservoir competence hosts, a prediction supported by empirical data and modelling simulations. Johnson & Thieltges [52] expanded the concept of the dilution hypothesis to include complex life cycle parasites. They reviewed the evidence for mechanisms through which community diversity and structure could influence disease, including parasite decoys, predators and low competency hosts. Though the generality of the dilution hypothesis is still debated [53–57], this negative relationship between parasitism and diversity seems especially clear in cases of biodiversity loss [58] but it is likely a scale-dependent phenomenon as are many other patterns [59,60]. At larger spatial scales of observation, one can find evidence for a positive relationship between diversity and parasitism [61,62]. Other factors like the transmission mode (density-dependent versus frequency transmitted or directly versus trophically transmitted) of the involved parasites [52,63] and the type of predation (intraguild versus non-intraguild predation) [64,65] have also been implicated to modulate the dilution effect, but are difficult to constrain for historical or fossil assemblages. Before we can analyze other factors, we document the dominant patterns on large timescales [66].

What then can we learn from the fossil record of parasitism through geologic time? Given the nature of the fossil record of parasite–host interactions, only rarely is it possible to ascertain the taxonomic identity of parasites precisely [30,33,67]. Therefore, it is nearly impossible to quantify parasite diversity, but it is possible to reconstruct relative changes in parasitism through geologic time in two alternative ways [68,69]. While we cannot directly test the amplification and dilution hypotheses in deep time in the same way as in living systems, we can test the relationships between the occurrence of parasitic traces (and the prevalence of parasitic traces) with the diversity of their hosts through the history of animal life. There is at least some support that diversity patterns in parasites might be closely linked to that of their hosts [70]. How have the number of occurrences of parasitism within geologic periods and the prevalence of parasitism changed since the first known occurrence in the early Cambrian? How are these variables related to host diversity throughout the Phanerozoic? We have compiled data from the published literature on fossil marine ectotherms displaying evidence of parasitic interactions and diversity indices of these hosts to test the following hypotheses:

(1) Parasitic interactions, as measured by the number of occurrences within a geologic time bin (period) and prevalence in species collections, have increased through the Phanerozoic.
(2) There is a positive association between host diversity and parasitic interactions over long evolutionary timescales (analogous to amplification) rather than a negative association (analogous to dilution).

## 2. Methods

We compiled data on the occurrences of parasite–host interactions as evidenced by parasitic remains or traces (including characteristic pathologies) in skeletons of Phanerozoic marine metazoan hosts from the published literature focusing primarily on, but not solely, invertebrates. Our database contains screened host remains, identified to the genus or species level, that show evidence of parasitic interactions in the form of characteristic traces (positive observations) as well as data on available co-occurring taxa that lack evidence for parasitic interactions. To meaningfully evaluate the occurrence (i.e. presence of traces) and prevalence (i.e. the proportion of affected individuals in a sample) of parasitism through Earth's history, we need a biologically meaningful definition of parasitism that can also be applied in the fossil record. Here we define parasitism as a long-term close interaction between individuals of two species wherein one benefits to the detriment of the other, though generally not resulting in the latter's death [32]. Irrespective of the identity of the culprits, comparisons with the behaviour of modern parasites with similar behaviour as well as population data of the impact on their hosts allow us to assess their impact on host populations and therefore infer a parasitic relationship even in now-extinct parasite–host associations [41,71–73]. We compiled all interactions which have been attributed to parasitism and assign certainty categories to them. The gold standard, our category 1, are interactions where a benefit for the parasite taxon can be plausibly demonstrated and a negative effect of infested host has been quantitatively demonstrated within host samples derived from a particular locality and stratigraphic unit. Category 2 refers to interactions preserved in the fossil record, where similar interactions involving the same parasite taxa have been shown to lead to negative impact today and/or in the past. Category 3 refers to interactions that show a clear negative impact on the individual host specimen consistent with our definition of parasitism but the culprit is unknown or a wide negative impact of this interaction still needs to be more widely modelled. Category 4 refers to interactions that could be consistent with parasitism but also other interactions and, therefore, are excluded from further analyses. The position and characteristic morphology of these parasitic remains and traces can indicate the type and behaviour of the parasites which also allows us to identify the culprit in multiple systems. Model systems include the gastropod–echinoderm, isopod–decapod and trematode–bivalve interactions [72,74–78]. Variables collected include the sample size of the occurrence, taxonomic data on hosts and parasites (when available), and lithostratigraphic and geochronological contexts of the samples. Only parasitism data from fossil occurrences and Holocene death assemblages (but not live-collected samples) were analysed in this study. Prevalence, the proportion of individuals bearing evidence of parasitism, was calculated for each occurrence comprising 10 or more (fossil) remains. Median prevalence values and bootstrapped 95% confidence intervals were calculated for each era.

Genus-level occurrence data for each host class were downloaded from the Paleobiology Database (PBDB) via the FossilWorks website (16 November 2020 for all groups except the Actinopteri and Anthozoa, which were downloaded on 25 and 22 January 2021, respectively). To minimize potential biases in constructing diversity curves, the occurrence data were sub-sampled using Alroy's [79,80] shareholder quorum sub-sampling (SQS). The sampling quorum per time interval (period) was 0.6 with 50 trials to calculate mean sampled diversity, three-timer (3T) origination rate and 3T extinction rate [79,80].

Two indices of parasitism were related to diversity data via generalized linear models (GLM): (i) the number of species (or genus) level occurrences of hosts showing evidence of parasitism per period, and (ii) species-level occurrence prevalence values. The number of occurrences per period was related to the three SQS diversity indices (mean sampled diversity, 3T origination rate and 3T extinction rate) and the midpoint age of the geologic period in millions of years ago (Ma) weighted by the log_10_-transformed number of specimens in a GLM using a Poisson link function. Individual prevalence values of host taxa with at least 10 specimens were related to the SQS diversity indices and best estimate age for each sample (Ma) weighted by the log_10_-transformed number of specimens in a GLM using a binomial link function.

All statistical analyses were conducted and figures assembled using *R* freeware (v. 4.0.3) and the following packages: *ggplot* (Wickham, 2016), *ggthemes* (Arnold, 2019), *dplyr* (Wickham *et al*., 2020), *rcompanion* (Mangiafico, 2020) and *viridis* (Garnier, 2018). *R* scripts are available in the electronic supplementary material. An *α*-value of 0.05 is assumed for statistical significance in all analyses and *p* < 0.10 is described as marginally significant in GLMs.

## 3. Results

**Figure 1.**
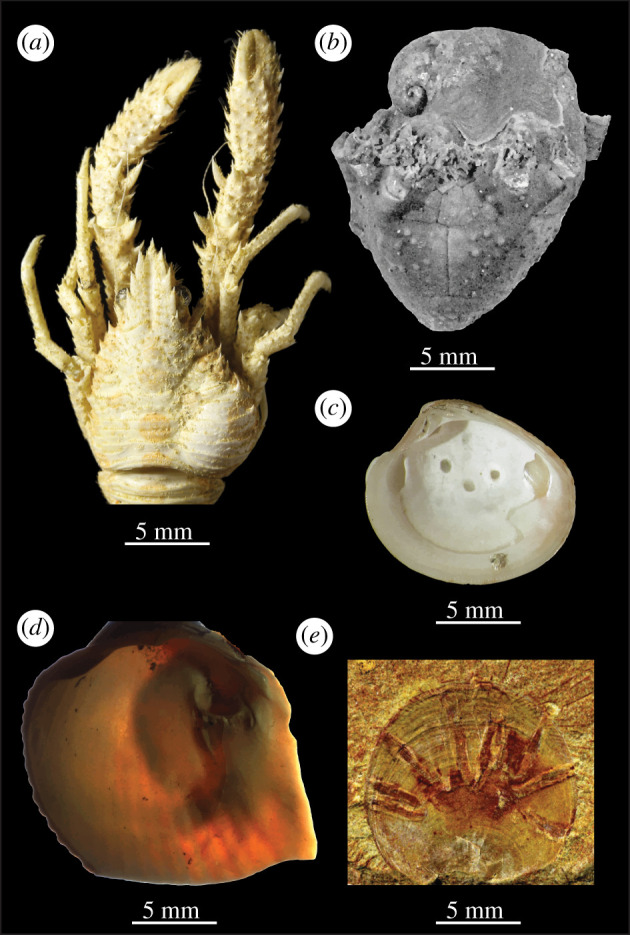
Examples of parasite–host interactions preserved on marine animal host skeletons. (*a*) Modern isopod-infested decapod (as indicated by swelling on right side), *Galathea* sp. Camiguin, Philippines (Klompmaker *et al*. [77]; Creative Commons Attribution License). (*b*) Middle Devonian crinoid, *Arthroacantha carpenteri*, infested by platyceratid gastropod (placed on the topmost side of the picture), Silica Shale, Sylvania, Ohio, US (Baumiller & Gahn [32]; reproduced with permission). (*c*) Holocene trematode-induced circular pits on interior of bivalve *Chamelea gallina*, specimen 129 from 13.10 m depth sample of core 240S8, Po River plain, Italy (photomicrograph by JWH). (*d*) Modern death assemblage spionid polychaete-induced mudblister (right side) on bivalve *Clinocardium nuttalli*, Monroe Landing, Whidbey Island, Washington, USA (transmitted and reflected light photomicrograph; JWH collections: photomicrograph by Gabriel S Jacobs). (*e*) Early Cambrian encrusting tubes of unknown kleptoparasite on the brachiopod *Neobolus wulongqingensis*, Guanshan Konservat-Lagerstatte, Wulongqing Formation, eastern Yunnan, China (Zhang *et al*. [41]; Creative Commons Attribution 4.0 International License).

**Figure 2.**
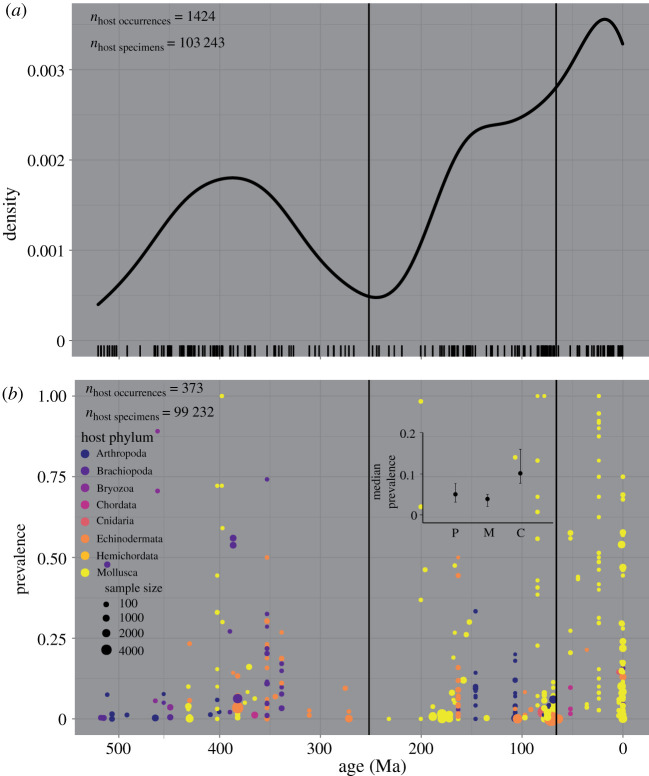
Phanerozoic history of parasitism occurrence and prevalence among marine animals. (*a*) Rug plot (along *x*-axis) and density plot of the temporal distribution of single host taxon occurrences through geologic time. (*b*) Prevalence values of occurrences with at least 10 specimens through geologic time colour-coded by host phylum. Inset plot, median prevalence values by era.

The compiled dataset contains 2118 observations (species-level occurrences) of biotic interactions reported to be parasitism, ranging in age from Cambrian to Holocene. Evidence for parasitism occasionally derives from parasites being preserved *in situ* on hosts but most evidence is in the form of a variety of borings, pits, blisters, pearls, growth responses and other malformations preserved on hosts (figure 1; electronic supplementary material, table S1). We interpret that 1424 of the 2118 observations unambiguously represent parasitism (categories 1–3 as defined above) as they coincide with morphological evidence for a clear negative impact on their host during life and a benefit for the parasitic organism based on their position, orientation and mode of life. Host phyla include Arthropoda (17.8% [72,77,78,81–85]), Brachiopoda (4.8% [38,39,41,86–93]), Bryozoa (2.0% [94–97]), Chordata (1.9% [98–101]), Cnidaria (2.7% [102–108]), Echinodermata (21.2% [75,109–117]), Hemichordata (1.9% [118]), Mollusca (47.3% [76,77,119–133]) and Porifera (less than 1% [134,135]). Parasite phyla include Annelida (12.1% [123,136,137]), Arthropoda (17.3% [72,77,78,113,116,117,138–140]), Brachiopoda (less than 1% [141]), Bryozoa (1.0% [142]), Cnidaria (1.2% [143]), Echinodermata (less than 1%), Foraminifera (less than 1% [144,145]), Mollusca (14.2% [109,146–149]), Nematoda (less than 1% [81]), Phoronida (less than 1% [150]), Platyhelminthes (11.5% [76,151–153]), Porifera (1.8%) and parasites of unknown taxonomic affinity (39.0% [154]). There are peaks in the density of parasitism occurrences among the three Phanerozoic eras occurring in the Devonian, Jurassic and Neogene periods, respectively (figure 2).

Prevalence values were calculated for 373 single species occurrences whose sample sizes were 10 or greater, representing 99 232 specimens. Prevalence values ranged from 0 to 1 with mean and median values of 0.16 and 0.06, respectively. Median prevalence values were 0.05 or 5% for the Paleozoic (*n* = 112), 0.04 or 4% for the Mesozoic (*n* = 144) and 0.10 or 10% for the Cenozoic (*n* = 117). Median prevalence values for the Paleozoic and Mesozoic were statistically indistinguishable from one another (*p*_Mann–Whitney_ = 0.19) but the Cenozoic value was significantly greater than the first two (*p*_Mann–Whitney_ = 0.00017 versus Paleozoic and *p*_Mann–Whitney_ = 1.055 × 10^−07^ versus Mesozoic; figure 2 inset). This temporal pattern was robust to only using the 311 singles species occurrences where sizes were 20 or greater, representing 98 382 specimens: Paleozoic median prevalence = 0.04, Mesozoic median prevalence = 0.03 and Cenozoic median prevalence = 0.07 (*p*_Mann–Whitney_ = 0.16 Paleozoic versus Mesozoic; *p*_Mann–Whitney_ = 0.02 Paleozoic versus Cenozoic; *p*_Mann–Whitney_ = 5.31×10^−5^ Mesozoic versus Cenozoic).

The results of the GLMs comparing the number of species-level occurrences of parasitism per period to SQS mean sampled diversity values, SQS 3T origination rates, SQS 3T extinction rates and the median age of the geologic time period are presented in tables 1 and 2 (electronic supplementary material, table S2). The GLM including data for all host classes resulted in positive coefficients correlating occurrences to mean sampled diversity (*p* < 0.001) and origination rates (*p* < 0.01) and negative coefficients correlating occurrences to extinction rates (*p* < 0.001) and geologic age (*p* < 0.001). Similarly, parasitism occurrences are significantly and positively correlated to mean sampled diversity for stalked echinoderms (crinoids and blastoids), molluscs in general and cephalopods, specifically. Conversely, parasitism occurrences and mean sampled diversity are significantly and negatively correlated for arthropods and echinoids. Negative and marginally significant (*p* < 0.10) relationships between parasitism occurrences and mean standing diversity were found for cnidarians and bivalves. The GLMs for Brachiopoda (Linguliformea + Rhynchonelliformea) revealed no significant correlations between parasitism occurrences and the other variables (table 1).

**Table 1.** Results of generalized linear models for occurrence of infected hosts binned by period.

host phylum	host class	SQS mean sampled diversity	SQS 3T origination rate	SQS 3T extinction rate	midpoint age (Ma)
all	—	0.0029***	0.0842**	−0.3335***	−0.0013***
Arthropoda	—	−0.0178***	1.0185***	−0.4812*	−0.0022*
Brachiopoda	—	−0.0126	1.4809	0.4522	0.0085
Cnidaria	Anthozoa	−0.2329#	−0.2205	6.4477#	−0.0044
Echinodermata	—	0.0019	−1.0181***	0.3450***	0.0074***
—	Echinoidea	−0.9116***	8.0950***	10.6561***	—
—	Crinoidea and Blastoidea	0.0237***	−0.3511***	−0.1124	—
Mollusca	—	0.0126***	−0.1033*	−0.4983***	−0.0020***
—	Bivalvia	−0.0188#	−0.5956	3.3853**	−0.0209***
—	Cephalopoda	0.0434***	−0.9186***	−1.2428***	—

#*p*<0.10, *p<0.05, **p<0.01, ***p<0.001 (#, GLM not weighted by the number of individuals due to inadequate sample size data available).

**Table 2.** Summary of evidence for the dilution hypothesis and amplification hypothesis by the occurrence of infected hosts binned by period. Italic text indicates the primary mechanism in a given host phylum or class.

host phylum	host class	dilution	amplification	mechanism
all	—		supported	(+) origination rate; (−) *extinction rate*; minor (−) age influence
Arthropoda	—	supported		(+) *origination rate*; (−) extinction rate; minor (−) age influence
Brachiopoda	—	?	?	no significant relationships
Cnidaria	Anthozoa	marginally supported		marginal (+) extinction rate
Echinodermata	—		supported	(−) *origination rate*; (+) extinction rate; minor (+) age influence
—	Echinoidea	supported		(+) origination rate and (+) extinction rate
—	Crinoidea and Blastoidea		supported	(−) origination rate
Mollusca	—		supported	(−) origination rate; (−) *extinction rate*; minor (−) age influence
—	Bivalvia	supported		(+) extinction rate; minor (−) age influence
—	Cephalopoda		supported	(−) origination rate; (−) *extinction rate*

The results of the second set of GLMs comparing the prevalence values of individual species-level occurrences of parasitism to the period-level SQS mean sampled diversity, 3T origination rate, and 3T extinction rate, and age of the sample are available in tables 3 and 4. When considering all taxa, prevalence is significantly and positively correlated to mean sampled diversity and significantly and negatively correlated to origination rates and extinction rates. Among Mollusca, there is a significant negative correlation between parasite prevalence and extinction rates.

**Table 3.** Results of generalized linear models for species occurrence level prevalence values of infected hosts versus diversity values and age weighted by sample size.

host phylum	host class	SQS mean sampled diversity	SQS 3T origination rate	SQS 3T extinction rate	best age (Myr)
all	—	0.0112**	−0.4600*	−0.7476**	0.0015
Brachiopoda	—	−0.1529	−8.8259	—	0.0591
Echinodermata	—	−0.0234	−0.1048	0.0479	0.0088
—	Echinoidea	1.0390	–	–	0.0472
—	Crinoidea and Blastoidea	−0.0882	−1.8123	1.4897	0.0254
Mollusca	—	0.0185	−0.4793	−1.0626**	0.0008
—	Bivalvia	−0.0633	2.0956	−0.2988	−0.0270
—	Cephalopoda	0.1233#	−1.9609	1.2845	+0.0438**

#*p*<0.10, *p<0.05, **p<0.01, ***p<0.001.

**Table 4.** Summary of evidence for the dilution hypothesis and amplification hypothesis by species occurrence level prevalence values of infected hosts versus diversity values of host class by period. Italic text indicates the primary mechanism in a given host phylum or class.

host phylum	host class	dilution	amplification	mechanism
all	—		supported	(−) origination rate; (−) *extinction rate*
Brachiopoda	—	?	?	no significant relationships
Echinodermata	—	?	?	no significant relationships
—	Echinoidea	?	?	no significant relationships
—	Crinoidea and Blastoidea	?	?	no significant relationships
Mollusca	—		supported	(−) extinction rate
—	Bivalvia	?	?	no significant relationships
—		Cephalopoda	marginally supported	minor (+) age influence

## 4. Discussion

### (a) Increase of parasitism through time

It is reasonable to assume that parasitism has become more severe since its first occurrence on an animal host, sometime between the terminal Ediacaran to early Cambrian periods, but, until now, few studies have systematically and quantitatively addressed this assumption. Vermeij [46,155] proposed the hypothesis of escalation, which states that enemies (predators, parasites, dangerous prey, competitors, etc.) are likely the primary agents of natural selection that influence macroevolutionary patterns. Our analyses demonstrate an increase in parasite–host interactions throughout the Phanerozoic (figure 2*a*). When considering all 1424 occurrences of parasitism in the compilations, we see a step-wise increase in the number of occurrences among the three eras of the Phanerozoic Eon. This increase is even starker as an increase in occurrences per era corresponds with a decrease in temporal duration of the same eras. Specifically, 481 occurrences over the 289 million years (Myr) of the Palaeozoic (1.66 Myr^−1^), 492 occurrences over the 185 Myr of the Mesozoic (2.66 Myr^−1^) and 451 occurrences over the 66 Myr of the Cenozoic (6.83 Myr^−1^). One caveat to a strictly biological interpretation of this pattern is the first-order prediction that taphonomic processes have reduced the quality of preservation with age [156,157], though this is not always the case [158,159]. For example, molluscs preserved in geologically younger non-lithified sediments are easily extracted and examined for parasite-induced traces. By contrast, older specimens are often preserved in, as well infilled by, lithified sediment, which impedes thorough examination. Moreover, original shell material often dissolves leaving only an internal mould, though such fossil preservation has provided evidence for parasitic interactions as far back as the Silurian [76,120,137].

Prevalence values (figure 2*b*) provide more insight into the ecological importance of parasitism than occurrence values alone because they are calculated as a proportion of the sample and are less prone to taphonomic or sampling heterogeneities. Only 26% of the observations in our compilation reported sample sizes of 10 or more, allowing us to calculate a prevalence value. This could be because instances of parasitism have often been seen as an oddity or their description was not the primary purpose of the research. Nevertheless, we were able to construct an unprecedented record of parasite prevalence among marine invertebrates with observations from the Paleozoic (*n* = 112, 0.39 Myr^−1^), Mesozoic (*n* = 144, 0.78 Myr^−1^) and Cenozoic (*n* = 117, 1.77 Myr^−1^). Similar to the number of occurrences through time, prevalence values indicate an increase of parasitism throughout the Phanerozoic, though with a difference in timing. The median prevalence value for the Cenozoic was significantly higher than the statistically indistinguishable median prevalence values of the preceding eras (figure 2*b* inset). We hypothesize that the Phanerozoic history of parasite-induced traces among marine animal hosts reflects an escalation of parasite–host interactions (figure 2). This same time interval witnessed numerous mass extinctions, evolutionary radiations and biotic turnover across a variety of temporal and spatial scales. Next, we will examine the relationship between parasitism and diversity at course temporal binning over the last 540 Myr.

### (b) Relationships between parasitism and diversity

There is ample evidence that metazoan biodiversity has waxed and waned through the last 541 Myr, though the nature of the overall pattern has been extensively debated [79,160–163]. Though incomplete in preservation and sampling to varying degrees through time, the fossil record provides physical evidence for ancient life that would not otherwise have been known from the evolutionary analysis of living clades. Sepkoski's [23,162] estimates of diversity through time were based on a compilation of the first and last occurrences of marine animal genera and the assumption that each ranged through their entire interval. While it is not unreasonable to assume that these genera existed between their first and last occurrences, including taxon occurrences in time bins from which they have not been sampled results in a variety of problematic biases when constructing diversity curves through geologic time [80]. For this reason, we used Alroy's [79,80] shareholder quorum sub-sampling procedure on genus occurrence data for each host class derived from the PBDB to produce diversity estimates that include a fairer representation of uncommon genera.

#### (i) Evidence for the amplification hypothesis

The significant, positive relationship between mean sampled diversity and the number of parasitism occurrences for all taxa and prevalence is consistent with the amplification hypothesis (tables 2 and 4). The number of parasitism occurrences and prevalence is also significantly and negatively correlated to extinction rates, suggesting that extinction suppressed parasitism, though, without data on parasite diversity, these results do not allow us to distinguish between co-extinction of parasites and hosts or merely the extinction of hosts. Origination rates have a more complex relationship with parasite occurrence and prevalence. The occurrence of parasite hosts increases with host origination rates, supporting amplification, but prevalence values decrease, perhaps suggesting that even though parasitism is becoming more common as host diversity increases, its prevalence decreases, consistent with dilution. Consistent with the results presented in figure 2, the occurrence of parasitism strongly increased through geologic time. These pooled results include a variety of phyla and classes with very different body plans, life modes, parasitic interactions and proportional representation in the dataset, so it is beneficial to dissect the data into more finely resolved taxonomic groups.

The consistent positive relationship between both indicators of parasitism and extinction rate for molluscs is striking for several reasons (tables 2 and 4). First, SQS measures of diversity, origination rate and extinction rate do not suffer from the same taphonomic factors and limitations of Sepkoski's [23] compilation and are currently among the best proxies for constructing relative changes in bivalve biodiversity through the Phanerozoic at period-scale of observation. The second is that the prevalence of parasitism within a sample is not subject to the same factors related to differences in sampling effort or availability as counting the number of occurrences of parasitism within a bin. The consistent positive relationship between parasitism and extinction rate of mollusc hosts, which make up approximately 47% of host occurrences and approximately 58% of prevalence values, is likely a robust pattern (table 3). As with Huntley & Kowalewski [14], interpreting the meaning of this pattern can be more tricky. The prevalence of parasitism in all examined taxa is mostly low (less than 1–5%), although they can likely reach very high numbers in specialist parasites or particular environmental circumstances [119]. One can imagine a scenario similar to escalation [155], the Red Queen [164] or other hypotheses [165] where parasitism acts as a selective force that promotes the evolution of their hosts [27,120,165]. Alternatively, as biodiversity generally increases, new life modes and biotic interactions, parasitism included, are likely to evolve; a passive increase in ecological life modes with increasing diversity.

**Figure 3.**
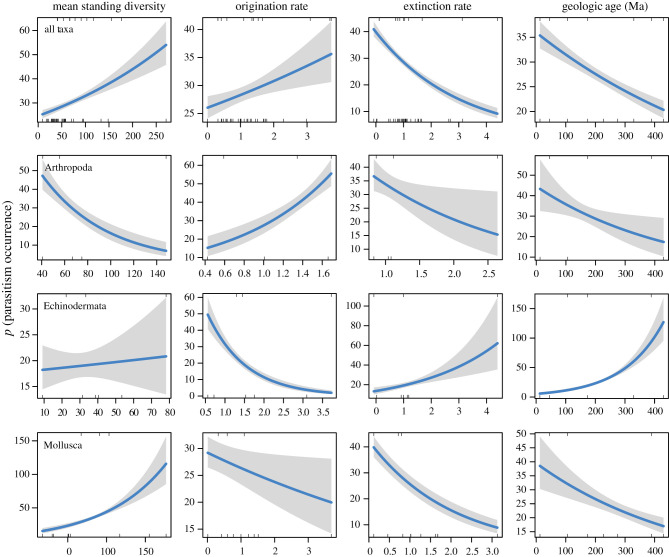
Graphical depiction of GLM of occurrences (*y*-axes) versus SQS mean standing diversity, SQS 3T origination rates, 3T extinction rates, and midpoint age of geologic time period (as reported in table 1). The solid line depicts the resulting generalized linear model and the grey field represents its 95% confidence interval. (Online version in colour.)

**Figure 4.**
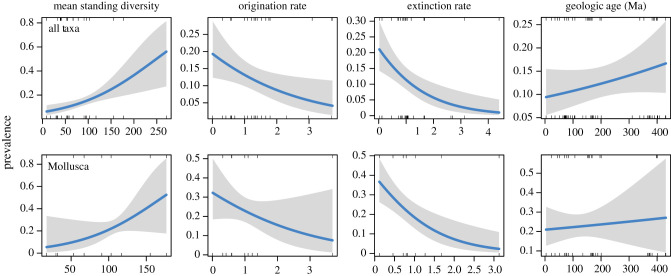
Graphical depiction of GLM of prevalence values (*y*-axes) versus SQS mean standing diversity, SQS 3T origination rates, SQS 3T extinction rates and best estimate age of sample (as reported in table 3). The solid line depicts the resulting generalized linear model and the grey field represents its 95% confidence interval. (Online version in colour.)

On longer timescales, an increase of parasitism might at first glance be the dominant factor as there is good evidence for a step-wise increase of modern groups of marine parasites [28] with some appearing in the Paleozoic [137,166], the Mesozoic [167] or Cenozoic [102]. This would also be consistent with the positive correlation with origination rates (tables 1 and 3). However, there is also support for the extinction of specialized parasite–host associations in the past [69,115,154,168] and it has been argued that co-extinction with hosts might be an important driver of extinction for parasites and symbionts more generally [169–173]. An important role of diversity loss has also been postulated for the modern dilution effect hypothesis [58]. We cannot entirely rule out that changes in both diversity and parasitism are being affected by an independent process such as sample availability, though, again, this is unlikely given that the pattern holds for prevalence and the fact that it is robust against possible biases produced by differences in sample size (figures 3 and 4; tables 1 and 2).

Our results (tables 2 and 4) seem to indicate that the dominating mechanisms might differ even within the same phylum with the class Cephalopoda being consistent with the results obtained for Mollusca as a whole, while the class Bivalvia rather seems to support dilution by showing a marginally significant negative correlation with diversity and a positive correlation with extinction rates at least for occurrences.

The occurrences of parasitism in crinoids and blastoids might also support the amplification hypothesis, though likely through a different mechanism than that controlling molluscs. These stalked echinoderms display significant negative relationships between parasite occurrence and origination rates, which results in a positive relationship with mean sampled diversity indicated by a positive coefficient an order of magnitude smaller than those of the evolutionary rates. One difference between the stalked echinoderms and molluscs is the predominant type of parasitism for each group. Crinoids and blastoids were typically infested by external parasites (such as platyceratid gastropods) or gall-forming parasites boring into the stems from the outside [32]. Molluscs, on the other hand, often suffered from parasites infesting their viscera [76,87,119,120] with the occasional external kleptoparasites [41]. Their different types of parasites would have rather different transmission modes and would also be affected by predation differently, but it is difficult to predict from the fossil record alone. Platyceratid gastropods, for example, might have spent a large part of their life on a single host and there is good evidence that they might have been specifically targeted by ‘non-intraguild’ predators rather than their hosts [20]. It is tempting to attribute the amplification to the density-dependent mode of transmission as the non-intraguild predation should have diluted rather than amplified its effects. In the case of internal parasites with complex life cycles, increased intraguild predation might be a possible explanation for their amplification with diversity as frequency-dependent transmission is expected to lead to a dilution effect. Peculiarly, there is some support for the dilution effect in bivalve molluscs when looking at occurrences of parasitism but this does not seem to hold when looking at prevalences of parasitism. It should be noted in this context that the raw median prevalence values are lower in the Paleozoic and Mesozoic than in the Cenozoic for bivalves, which would be consistent with amplification but these do not seem to hold up when weighting for sample size which is an order of magnitude greater in the Cenozoic than in the Paleozoic and Mesozoic. This highlights that more work is necessary to understand the impact of particular mechanisms and as our analyses focus on large-scale temporal scales—where amplification might be the dominant model as our results suggest.

#### (ii) Evidence for the dilution hypothesis

Contrary to our initial hypothesis, we also found evidence supporting a significant negative relationship between parasitism occurrences and mean sampled diversity in arthropods (mainly decapods approx. 81% and trilobites approx. 19%; tables 1 and 2) and echinoids as well as marginally in bivalves and Anthozoa, but varying relationships with origination and extinction rates. In arthropod and echinoid hosts, dilution of parasitic interactions is associated with the increase in origination rate, but they are variably related with extinction rate. In anthozoan, bivalve and echinoid hosts, parasitism is associated with a positive association with extinction rate, while it is associated with a negative correlation with extinction rate for arthropods. These are organisms with drastically different body plans, life modes and means of securing nutrition. Generalizations of parasitic interactions in these groups are more difficult to make as their impacts (e.g. disease) are dependent on environmental conditions [174–176] as well as modulation by non-intraguild predation and density are not directly studied or complex [25,177], which makes them even harder to predict for past interactions and our scale of analysis. More prevalence data for these groups are necessary to better establish the mechanisms behind these differences.

### (c) Closing thoughts and future prospects

We have presented the first synthesis of marine parasite–host interactions among 10 host phyla and at least 13 parasite phyla across the Phanerozoic. The counts of occurrences and prevalence values among individual samples indicate an increase of parasitism over the last 541 Myr. Comparisons of the fossil record of parasitism with SQS estimates of host mean sampled diversity, three-timer origination rates and three-timer extinction rates reveal significant correlations that primarily support the amplification hypothesis. For all taxa pooled, we find significant, positive correlations between diversity and parasitism, and origination rate and parasitism; and significantly negative correlations between extinction rate and parasitism, regardless of the proxy used for parasitism. The most consistent of these relationships on the phylum-level are found among mollusc hosts with a negative correlation with extinction rate.

This work represents a sizable step in establishing the Phanerozoic pattern of parasitism and a step toward understanding the processes relating parasitism with diversity across the broad history of marine animal life. Admittedly, we have used large temporal bins in these initial analyses. This approach was necessary to maintain a reasonable number of observations per bin. We are seeking to increase temporal resolution in ongoing and future analyses of our expanding dataset. Additionally, we aim to incorporate climate and environmental proxies as well as data related to parasite transmission mode and impact of predation in our models to assess the roles of abiotic and other biotic factors. We hope that researchers will gain more interest in not only screening their fossils for signs of parasitism but also reporting the numbers of individuals bearing evidence of parasite–host interactions, the total numbers of individuals in the examined samples, and comparable information for samples in which no evidence of parasitism was found. This will allow us to better understand the mechanisms driving changes in parasite prevalence [33,178], and modelling might contribute to further understanding the patterns once larger datasets become available [179]. We have shown after spending much of the last decade investigating parasite–host interactions among a diverse group of marine invertebrates that these traces are much more common in the fossil record than we previously knew. It is likely that many more discoveries to be made will provide important insights on the links between ecology, life history and environmental factors in driving the evolution of parasite–host associations.

## Data accessibility

Data to reproduce our study are provided in the electronic supplementary material as .csv files [180].
